# Lanthanide Nanotheranostics in Radiotherapy

**DOI:** 10.3390/ijms27010426

**Published:** 2025-12-31

**Authors:** Shaofeng Han, Yurun Liu, Taoyang Cai, Yanru Liu, Shangjie Ge-Zhang

**Affiliations:** 1 School of Energy Power and Mechanical Engineering, North China Electric Power University, Beijing 102206, China; shaofenghan@ncepu.edu.cn (S.H.);; 2 College of Science, Northeast Forestry University, Harbin 150040, China; 3 College of Pharmacy, Guizhou University of Traditional Chinese Medicine, Guiyang 550025, China

**Keywords:** lanthanide nanomaterials, radiosensitizer, tumor radiotherapy, reactive oxygen species, review

## Abstract

Radiotherapy, a cornerstone of cancer treatment, is critically limited by tumor radioresistance and off-target toxicity. Lanthanide-based nanomaterials (Ln-NPs) have recently emerged as a versatile and promising class of theranostic radiosensitizers to overcome these hurdles. This review comprehensively outlines the state-of-the-art in Ln-NP-enabled radiotherapy, beginning with their fundamental physicochemical properties and synthesis and then delving into the multi-level mechanisms of radiosensitization, including high-Z element-mediated physical dose amplification, catalytic generation of reactive oxygen species (ROS), and disruption of DNA damage repair pathways. The unique capacity of certain Ln-NPs to serve as MRI contrast agents is highlighted as the foundation for image-guided, dose-painting radiotherapy. We critically summarize the preclinical and clinical progress of representative systems, benchmarking them against other high-Z nanomaterials. Finally, this work discusses the ongoing challenges, such as biocompatibility, targeted delivery, and regulatory hurdles, and envisages future directions, including combinatorial strategies with immunotherapy and the development of personalized nanotheranostic paradigms. Through this synthesis, this review aims to provide a clear roadmap for the continued development and clinical integration of lanthanide nanotheranostics in oncology.

## 1. Introduction

Radiotherapy is one of the cornerstones in the contemporary oncologic care, which can induce DNA breakage of tumor cells by high-energy rays, thus realizing the elimination of malignant tissues. However, the curative effect of traditional radiotherapy is often severely restricted by the inherent or acquired radiation resistance of tumor cells. At the same time, increasing the radiation dose to enhance the lethality will inevitably aggravate the damage of surrounding normal tissues. This fundamental contradiction between “dose-benefit” and “dose-toxicity” has seriously suppressed the promotion of therapeutic index. In order to break through this bottleneck, researchers put forward and extensively explored the strategy of radiosensitizer. Radiosensitizer is a kind of substance that can selectively improve the sensitivity of tumor tissue to radiation. Its core mechanism includes improving tumor hypoxia, inducing tumor cells to be in a more vulnerable cell cycle stage, inhibiting DNA damage repair pathway, and enhancing the deposition of radiation dose in tumor [1,2]. The selectivity of sensitizers can be divided into actively targeting tumor-specific molecules or receptors, and “passively” relying on tumor-specific enhanced osmotic and retention effect (EPR) or low pH/hypoxia microenvironment. In addition, the introduction of nanotechnology, especially the design based on metal nanoparticles with high atomic number, has effectively enhanced the deposition of radiation dose in tumors, while significantly reducing the accumulation and toxicity to normal tissues [3,4]. Furthermore, radiotherapy can also promote anti-tumor immunity by activating immune pathways in tumor microenvironment (for example, cGAS-STING induces type I interferon response), thus providing a theoretical basis for the combined application of sensitizer and immunotherapy [5,6]. Therefore, the introduction of sensitizer into radiotherapy scheme is not only expected to improve the radiation response of tumor cells, so as to achieve the same or better killing effect at a lower dose, but also provides a new path for reducing normal tissue damage and improving treatment safety and prognosis.

Among many radiosensitization strategies, sensitizers based on nanotechnology are gradually becoming a research hotspot. With its unique physical and chemical properties, nanoparticles can enhance the radiotherapy effect through many mechanisms. For example, nano-sensitizers based on materials with high atomic number (high-Z) can significantly enhance the interaction between rays and substances, promote secondary electron/radiation deposition, and thus expand tumor dose deposition [7,8,9]. Among these nanomaterials, lanthanide nanomaterials (that is, containing lanthanide elements: Z = 57–71) have been considered as a promising new tumor radiosensitizer because of their high atomic number, rich 4f electronic configuration and potential imaging/theranostic function. Lanthanide-based nanoparticles not only have advantages in physical aspects, such as X-ray absorption cross section enhancement, but also show strong adjustability and functional integration in surface modification, targeted delivery and tumor microenvironment response [9]. Compared with traditional small molecule sensitizers, lanthanide nano-materials show unique advantages in enhancing the interaction between rays and substances, promoting the production of tumor toxic substances and combining with diagnosis and treatment functions [10,11].

**Figure 1:**
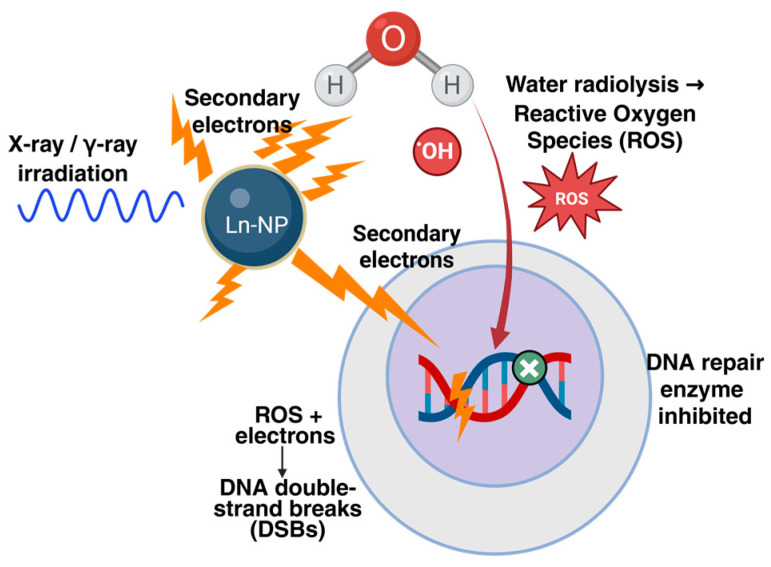
Mechanistic overview of radiation–lanthanide nanoparticle interactions. High-Z lanthanide nanoparticles absorb incident X-rays and emit secondary electrons, resulting in local dose amplification. These electrons induce water radiolysis to generate reactive oxygen species (ROS), which cause oxidative stress and DNA double-strand breaks. The excessive damage overwhelms DNA repair pathways (NHEJ/HR), ultimately enhancing tumour radiosensitisation. Additional mechanisms, such as hypoxia alleviation and X-ray–induced photodynamic therapy (X-PDT), may further potentiate this effect. Created in BioRender. Liu, Y. (2025) https://BioRender.com/gzhjj6p, accessed on 21 November 2025.

Ln-NPs exhibit a unique combination of properties, including a high atomic number that localizes dose, tunable redox activity for catalytic reactions, and intrinsic imaging capabilities, enabling them to act as potent, imageable radiosensitizers via multifaceted mechanisms. These integrated mechanisms, spanning physical, chemical, and biological levels, will be detailed in Section 3 [12,13,14]. Figure 1 schematically illustrates the mechanistic framework of radiation–nanoparticle interaction, summarizing the cascade from X-ray energy deposition and electron emission to ROS generation, DNA damage, and repair inhibition within tumor cells. In addition to sensitization, lanthanide nanomaterials also show the potential of Theranostics. Lanthanide nano-materials represented by gadolinium-based nanoparticles AGuIX can not only be used as effective radiotherapy sensitizers, but also have excellent image contrast ability, thus achieving accurate radiotherapy enhancement under image guidance. Phase I studies have shown that the nanoparticles can be clearly displayed in magnetic resonance imaging (MRI) after intravenous injection of AGuIX in patients with brain metastases, and there is no obvious new toxicity when combined with conventional whole brain radiotherapy. In a recent phase II study of Nano GBM, the widespread spread of AGuIX in glioblastoma (GBM) was also confirmed, showing its good in vivo permeability and tumor accumulation characteristics [15]. In addition, lanthanide-noble metal composite nanostructures such as GdO@aucore-shell nanoparticles have achieved synergistic optimization in terms of radiation energy absorption and reactive oxygen species (ROS) yield by combining the high Z characteristics of the gadolinium system with the plasma resonance enhancement effect of precious metals such as gold [16,17,18]. 

This kind of mixed metal-based nano-sensitizer not only improves the biological distribution and tumor penetration, but also significantly improves the accumulation in tumor and prolongs the residence time by designing targeted ligands and functional modification, thus enhancing the radiosensitization effect [19]. 

In summary, lanthanide nanomaterials combine high-Z radiosensitisation with intrinsic MRI contrast. Their comprehensive performance is evaluated in the following sections.

## 2. Lanthanide Nanomaterials Fundamentals

This chapter introduces the basic knowledge of lanthanide nanomaterials used for tumor radiotherapy sensitization, including the key properties of lanthanide elements, preparation methods and characterization methods of nanomaterials. These basic elements lay the foundation for understanding its sensitization mechanism and application effect.

### 2.1. Properties of Lanthanide Elements

Lanthanide elements, characterized by their partially filled 4f orbitals, possess a suite of distinctive optical, magnetic, and chemical properties arising from their electronic configurations [20]. These include sharp emission lines, long luminescence lifetimes, and paramagnetism, which form the foundation for their use in imaging probes [21]. Their chemical behavior and coordination flexibility further allow for stable integration into various nanomaterial matrices [22,23]. While specific properties like high atomic number and redox activity are crucial for their function as radiosensitizers, the detailed discussion of these application-driven mechanisms is consolidated in Section 3.

### 2.2. Synthesis of Lanthanide Nanomaterials

**Figure 2:**
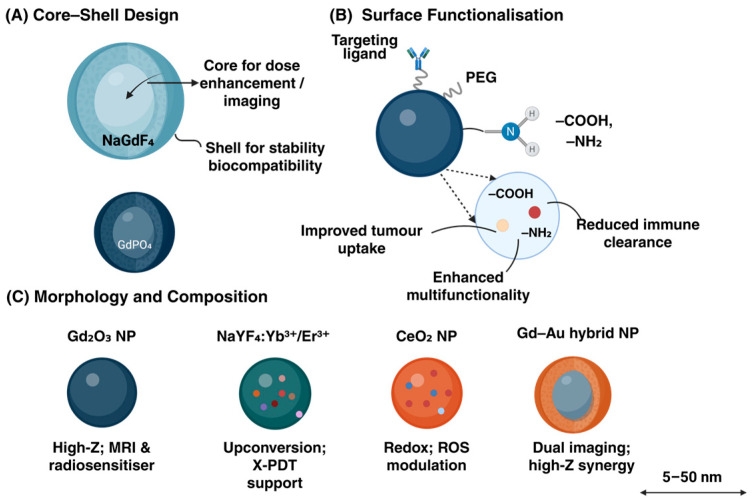
Structural design and diversity of lanthanide-based nanoparticles. (**A**) Core–shell design illustrating representative architectures such as NaGdF_4_@NaYF_4_ and GdPO_4_@Au. The core composed of high-Z lanthanide materials enhances X-ray absorption and MRI contrast, while the outer shell provides surface passivation, biocompatibility, and chemical stability; (**B**) Surface functionalisation demonstrating the attachment of PEG chains, targeting ligands (antibody/peptide), and conjugated radiosensitiser or drug molecules via –COOH and –NH_2_ functional groups. Such chemical engineering enables improved tumour uptake, reduced immune clearance, and enhanced multifunctionality; (**C**) Morphology and compositional diversity among lanthanide nanoparticles, including Gd_2_O_3_ (high-Z, MRI & radiosensitiser), NaYF_4_:Yb^3+^/Er^3+^ (upconversion, X-PDT support), CeO_2_ (redox-active, ROS modulation), and Gd–Au hybrid (dual imaging, high-Z synergy). Created in BioRender. Liu, Y. (2025) https://BioRender.com/gzhjj6p, accessed on 21 November 2025.

It is very important to prepare high-quality lanthanide nanomaterials for their excellent performance in tumor radiosensitization. Figure 2 illustrates the structural design and material diversity of lanthanide-based nanoparticles (Ln-NPs) used as radiosensitisers. Chemical wet methods, such as coprecipitation, hydrothermal method or solvothermal method, are still the mainstream processes, which are easy to operate and scale up, and the particle size and morphology can be easily controlled by reaction conditions, such as temperature, precursor concentration and ligand type [24,25]. For example, Gd_2_O_3_ nanoparticles prepared by solvothermal method show a highly crystalline and uniform morphology, which correspondingly enhances their photoelectric absorption and ROS generation efficiency, thus enhancing the sensitization effect [26]. Thus, wet-chemical routes readily yield such high-Z/plasmonic hybrids. At the same time, the recent research on the synthesis of lanthanide nanoparticles emphasizes that the structure-performance relationship of nanoparticles, such as crystallinity, particle size distribution, and surface ligand modification state, must be carefully regulated, which is particularly critical for building an integrated imaging-therapy platform [27]. From a more macroscopic design perspective, metal-based nanomaterials (including lanthanides) are an integrated system of diagnosis and treatment, and their synthesis methods, structure/surface modification and even biological removal behavior all determine their safety and functional performance, so the fine control of the synthesis stage is directly related to clinical feasibility [19]. In addition, in the image-guided radiotherapy system, particle size, dispersion state, hydration diameter (DLS), surface zeta potential and the connection of target ligands must be considered simultaneously when synthesizing, because these factors determine its distribution in vivo and the combined ability of tumor accumulation and treatment sensitization [28]. To sum up, the synthesis of nano-sensitizers is not only a matter of controlling particle size and morphology, but also a matter of optimizing the whole chain from structure to surface to function to biological behavior. Green, microfluidic and multi-component hybrid syntheses have recently been introduced and demonstrated improved batch-to-batch consistency.

### 2.3. Characterization of Lanthanide Nanomaterials

To fully exploit the potential of lanthanide nanomaterials in radiosensitization applications, a thorough understanding of their structure and properties is essential. Advanced characterization techniques not only reveal the microstructure, composition, and interaction with biological systems of nanoparticles but also provide a scientific basis for optimizing their design. Firstly, in terms of crystal structure and morphology, X-ray diffraction (XRD) analysis can determine the crystal phase, crystal quality, and lattice parameters of the particles, while high-resolution XRD or synchrotron XRD can further detect lattice defects and stress states—all of which have been proven to be key structural factors affecting the imaging-treatment integration capabilities of nanoparticles [9]. Scanning electron microscopy (SEM/TEM) can directly provide information on the morphology and particle size distribution at the nanoscale, and high-angle annular dark-field-STEM combined with EDS can assess the elemental localization and interface bonding of lanthanide and metals in complex nanostructures. Secondly, in terms of surface chemistry, X-ray photoelectron spectroscopy (XPS) and Fourier transform infrared spectroscopy (FTIR) are often used to identify the chemical groups and bonding modes on the surface of nanoparticles, while dynamic light scattering (DLS) and Zeta potential analysis provide information on the colloid particle size and surface charge, respectively—these parameters have been proven to directly affect the in vivo circulation, penetration, and tumor accumulation capabilities of nanomaterials [29]. Additionally, in terms of optical and electronic properties, lanthanide elements possess fluorescence emission characteristics due to 4f-4f electron transitions, and time-resolved fluorescence spectroscopy can be used to distinguish the optical behavior of internal and external lanthanide ions, while X-ray absorption spectroscopy (XANES/EXAFS) using synchrotron radiation can reveal the valence state changes and coordination environment of lanthanide elements. Literature indicates that precise characterization of the crystallinity, particle size, and surface state of nanoparticles is the foundation for ensuring their efficient functional integration in imaging and treatment [30]. By comprehensively applying the above characterization techniques, researchers can systematically understand the structure-performance relationship of lanthanide nanosensitizers: for example, if it is found through characterization that the crystal defect density of a certain particle is directly related to the secondary electron yield, then in the synthesis design, the crystal quality should be prioritized; or if it is observed that the tumor cell uptake rate increases after specific surface ligand modification, then this strategy can be extended to other systems. In summary, comprehensive structural, morphological, surface, and photoelectric property characterization provides a scientific guide for targeted improvement of lanthanide nanomaterials, enabling them to more accurately serve in radiotherapy sensitization and imaging-treatment integration applications.

## 3. Radiosensitization Mechanism

The exceptional radiosensitizing efficacy of lanthanide-based nanomaterials (Ln-NPs) stems from a coordinated cascade of physical, chemical, and biological processes initiated upon their interaction with ionizing radiation. This section synthesizes these multifaceted mechanisms into a coherent framework, detailing how Ln-NPs enhance radiotherapy from the initial energy deposition to ultimate tumor cell eradication and immune activation.

### 3.1. Physical Dose Enhancement

The foundational physical principle underlying Ln-NP radiosensitization is the high atomic number (Z) of their constituent elements, such as gadolinium (Gd, Z = 64) or ytterbium (Yb, Z = 70). In the clinical X-ray energy range, high-Z materials exhibit a significantly increased probability of photoelectric absorption [31]. This leads to the preferential deposition of incident radiation energy in the immediate vicinity of the nanoparticles, triggering the emission of a large number of low-energy secondary electrons (Auger and photo-electrons) and resulting in localized dose amplification at the nanoscale [32]. This effect is maximized when the K-edge absorption energy of the lanthanide element aligns with the peak of the medical accelerator’s bremsstrahlung spectrum, further boosting secondary electron yield. The resulting nanoscale concentration of energy deposition directly translates to the induction of complex, clustered DNA damage, such as double-strand breaks (DSBs), forming the physical basis for enhanced biological effectiveness without proportionally increasing dose to distant normal tissues [33].

### 3.2. Chemical Radiosensitization

Ln-NPs potentiate radiotherapy through chemical radiosensitization, primarily by augmenting water radiolysis to generate ROS. Furthermore, materials such as CeO_2_ NPs mediate the catalytic conversion of tumor-derived H_2_O_2_ into highly cytotoxic ·OH radicals. This significant elevation of intracellular oxidative stress is supported by experimental evidence, including comet assays showing a ≥2-fold increase in DNA damage. Upon irradiation, high-Z nanomaterials can enhance the radiolysis of water, generating ROS like hydroxyl radicals (·OH) and superoxide anions (O_2_·^−^). Furthermore, certain lanthanide oxides, most notably cerium oxide (CeO_2_), possess catalase- and peroxidase-like nanozyme activities [34]. Their reversible Ce^3+^/Ce^4+^ redox cycle allows them to catalytically convert tumor-abundant hydrogen peroxide (H_2_O_2_) into highly cytotoxic ·OH, especially in response to the tumor microenvironment [35]. This radiation-triggered or self-catalyzed ROS surge causes extensive oxidative damage to DNA, lipids, and proteins. Critically, Ln-NPs can sustain ROS production even after irradiation ceases (an afterglow effect), leading to prolonged oxidative stress that depletes cellular antioxidants like glutathione (GSH) and collapses the tumor cell’s redox defense system, thereby synergizing with radiation-induced damage [36].

### 3.3. Cellular and Molecular Biological Effects

#### 3.3.1. DNA Damage Amplification and Repair Inhibition

Ln-NPs exacerbate and prolong radiation-induced DNA damage. Studies consistently show the marked increase and persistence of γH2AX foci, a biomarker for DSBs, in cells treated with gadolinium-based nanoparticles and radiation [37,38,39]. This excessive, complex DNA damage overwhelms the cell’s repair machinery. DNA-repair interference is emerging; multiple studies report sustained γ-H2AX foci and down-regulation of BRCA1/RAD51 after Gd-NP + RT. The resultant failure to adequately repair DNA lesions forces cells into apoptosis or permanent senescence, directly translating to enhanced radiotherapeutic cell kill [40,41].

#### 3.3.2. Organelle Targeting and Cell Death Pathways

Surface-engineered Ln-NPs can be designed to localize to specific organelles, modulating cell death pathways [42,43]. For instance, mitochondrial-targeted nanoparticles can disrupt the electron transport chain, promote cytochrome c release, and initiate the intrinsic apoptotic cascade [42,44]. Nuclear-localized Ln-NPs (NLS-conjugated) have not yet shown superior DNA damage in vivo compared with cytoplasmic retention, and further validation is required before design optimization [45,46].

### 3.4. Tumor Microenvironment Modulation and Immunological Synergy

The hypoxic tumor microenvironment (TME) is a major cause of radioresistance. Notably, some Ln-NP systems (e.g., Gd-Bi bimetallic particles, CeO_2_ nanozymes) can maintain efficient ROS generation under hypoxia via oxygen-independent pathways, such as H_2_O_2_ activation, thereby preserving their sensitizing effect [47,48]. Moreover, radiotherapy combined with Ln-NPs can remodel the immunosuppressive TME. They promote immunogenic cell death (ICD), releasing damage-associated molecular patterns and tumor antigens that activate innate immune sensors like the cGAS-STING pathway [49,50]. This enhances dendritic cell maturation and effector T-cell infiltration. When combined with immune checkpoint inhibitors (e.g., anti-PD-1/PD-L1), this approach can amplify the local therapeutic effect and potentially induce systemic anti-tumor immunity, as evidenced by abscopal (distant) effects in preclinical models [51,52].

### 3.5. Radiotherapy Enhancement Effect

Based on the above mechanisms, lanthanide nanomaterials exhibit multi-dimensional enhancement of the therapeutic efficacy of radiotherapy at the macroscopic level. Firstly, from the perspective of dose physics enhancement, by selectively introducing high-Z nanosensitizers into the tumor, the absorbed dose in the tumor can be significantly increased while the dose in non-target tissues can be reduced under the same external irradiation dose. Monte Carlo simulations and animal experiments have confirmed that after adding gadolinium oxide nanoparticles, the dose absorption at the tumor site increases, while the dose in the surrounding normal tissues decreases [53,54]. This dose optimization directly improves the safety and efficacy of radiotherapy.

In the TME, the oxygen-dependence of ionizing-radiation-induced free-radical formation often leads to radioresistance. Emerging studies suggest that certain lanthanide-based nanoparticle systems may maintain ROS generation even under low-oxygen conditions, thereby restoring or enhancing tumor cell radiosensitivity [55]. For instance, nanoparticle platforms that catalyze ROS production or relieve hypoxia have been shown to enhance radiotherapeutic outcomes [56,57]. Although quantitative evidence remains limited, this strategy offers a promising route to overcome hypoxia-mediated radioresistance [58,59]. 

Finally, in terms of the integrated diagnosis and treatment platform and combined treatment strategies, the advantages of lanthanide nanomaterials have been further magnified. Taking AGuIX nanoparticles as an example, they possess both MRI contrast and radiotherapy sensitization functions [60]. In MRI-guided dose-painting radiotherapy, they achieve a higher tumor dose concentration and better therapeutic efficacy [61,62,63,64]. Moreover, the bimetallic or multifunctional design enhances the dose enhancement factor (DEF) through the high Z synergistic effect, while also having imaging and radiotherapy functions. In terms of targeted modification, nanoparticles functionalized with the RGD peptide motif have been shown to significantly increase tumor accumulation compared with non-targeted versions. For example, RGD-modified Gd_2_O_3_ nanoparticles exhibited markedly higher tumor uptake and prolonged circulation relative to their unmodified counterparts [65,66,67]. Notably, when lanthanide-based radiosensitizers are combined with immunotherapy, such as anti-PD-1/PD-L1 checkpoint inhibitors, a synergistic amplification of therapeutic effect can be observed [68,69]. For example, one study on Gd_2_O_3_ nanoparticles combined with radiotherapy reported enhanced immunogenic cell death and activation of the cGAS-STING pathway in a triple-negative breast cancer model, which suggests potential for a systemic (abscopal) immune response [70,71]. While some preclinical studies indicate that nanoparticles can enhance the combined effect of radiotherapy and immunotherapy by improving tumor delivery of immunomodulatory agents, increasing tumor antigen release, and modulating the tumor microenvironment, direct, quantitative comparisons between lanthanide-based nanoparticles and traditional radiotherapy combined with immunotherapy at the same doses are still limited. Nanoparticle-mediated strategies often involve different dosing schedules, variable immune checkpoint inhibitors, and diverse tumor models, making strict head-to-head comparisons challenging. As such, existing evidence should be viewed as trend-level and mechanism-supported rather than definitive clinical proof of superiority. To our knowledge, there are currently no published head-to-head comparative studies that directly evaluate lanthanide-based nanoparticles versus other high-Z radiosensitizers such as gold nanoparticles (Au-NPs) or hafnium dioxide (HfO_2_/NBTXR3) in combination with PD-1/PD-L1 immune checkpoint blockade under the same experimental conditions. The existing literature on nano-biomaterials in radio-immunotherapy primarily explores the synergistic potential of different nanoparticle platforms to enhance radiotherapy and immune responses; however, these studies often differ in model systems, radiation regimens, and immune endpoints, precluding formal quantitative comparison between distinct high-Z radiosensitizers [72].

Accordingly, while lanthanide nanoparticles and other high-Z materials have each been investigated as radiosensitizers and may influence immune mechanisms (e.g., enhanced immunogenic cell death and tumor microenvironment modulation), the current evidence base does not support definitive comparative conclusions regarding their relative effectiveness when combined with PD-1/PD-L1 blockade. Further carefully controlled studies would be required to quantitatively assess and compare these multimodal combinations across representative models.

Another investigation demonstrated that Gd-based nanoparticles in conjunction with checkpoint blockade significantly enhanced the radiation-induced abscopal effect [73]. These findings indicate that such nanoradiosensitizers may not only boost local tumour control but also engage systemic antitumour immunity, thereby potentially widening the therapeutic scope of radiotherapy. Current radiogenomics research is attempting to identify which patients are most likely to benefit from lanthanide-based nanosensitization, and accordingly adjust the dose of nanoparticles and combination therapy (Table 1).

## 4. Research Paradigm

In recent years, a variety of new paradigms and ideas for enhancing tumor radiotherapy using lanthanide nanomaterials have emerged. This chapter will focus on three aspects: pre-clinical research, clinical trial progress, and cross-sectional comparative studies. It will summarize the main achievements and characteristics of current research.

### 4.1. Preclinical Studies

**Figure 3:**
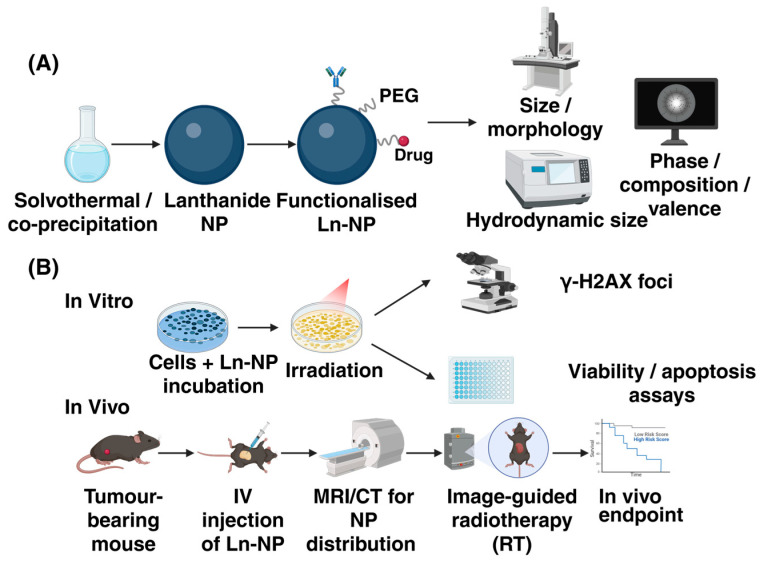
Experimental and workflow overview for lanthanide nanoparticle radiosensitization. (**A**) Synthetic and characterisation workflow: solvothermal or co-precipitation synthesis, functionalisation with PEG or therapeutic moieties, and physicochemical analyses (TEM, DLS, spectroscopic evaluation). (**B**) In-vitro and in-vivo experimental workflows: cellular incubation with Ln-NPs followed by irradiation and γ-H2AX or viability assays; tumour-bearing mouse models receiving intravenous Ln-NP injection, MRI/CT tracking, and image-guided radiotherapy (RT) for therapeutic evaluation. Created in BioRender. Liu, Y. (2025) https://BioRender.com/gzhjj6p, accessed on 21 November 2025.

To illustrate the overall experimental and evaluation process of lanthanide-based nanoradiosensitisers, Figure 3 summarises the representative workflow from synthesis and characterisation to in-vitro and in-vivo therapeutic assessment. This schematic highlights how physicochemical design, biological evaluation, and imaging-guided radiotherapy are integrated into a unified research framework.

A large number of in vitro cell experiments and animal model studies have systematically verified the effectiveness and mechanism of lanthanide-based nanomaterials as radiosensitizers. At the cellular level, multiple studies have demonstrated that the addition of gadolinium-based nanoparticles to irradiation significantly enhances markers of DNA damage compared with irradiation alone. For example, in triple-negative breast cancer cell lines (MDA-MB-231, MDA-MB-468), Sun et al. found markedly higher γ-H2AX foci counts in the AGuIX + X-ray group; meanwhile, colony-formation assays revealed that the combined treatment caused a substantial reduction in surviving cell colonies [75]. Similarly, a recent mechanistic review summarised in-vitro data wherein AGuIX roughly doubled the γ-H2AX foci after irradiation [37]. In animal xenograft models, the addition of AGuIX nanoparticles to radiotherapy has been shown to significantly improve tumour control. For example, in preclinical studies of AGuIX in hepatocellular carcinoma and soft-tissue sarcoma models, the nanoparticles combined with irradiation resulted in substantially greater tumour-volume reduction compared to irradiation alone [62]. Although specific numbers vary between models, these data provide strong in-vivo proof-of-concept that gadolinium-based nanoradiosensitizers can markedly enhance local tumour response to equal radiation doses.

Secondly, the research was further expanded to include different tumor models and various types of nanomaterials, in order to consolidate the aforementioned findings. For example, in animal studies AGuIX nanoparticles have shown tumour-to-normal-tissue uptake ratios of several-fold depending on model and timepoint, indicating substantial selective tumour accumulation [76,77]. A variant of AGuIX nanoparticles in which part of the Gd^3+^ ions were replaced by Bi^3+^ demonstrated enhanced radiotherapy efficacy in vivo and in vitro: increasing the Bi content was associated with greater DNA damage and improved tumour control, consistent with the expectation of a higher dose-enhancement factor (DEF) due to the higher atomic number of Bi compared with Gd. Although precise quantitative values vary, simulation studies indicate that Bi nanoparticles can achieve higher DEFs than Gd alone [78,79], and that lanthanide- or other high-Z nanoparticle radiosensitizers can enable a “dose-painting” paradigm in radiotherapy by increasing tumour dose deposition (via nanoparticle uptake) while reducing or sparing normal tissue exposure, and several Monte Carlo studies highlight the potential for significantly improved therapeutic ratio [80,81,82].

However, the previous clinical studies also pointed out several issues that need to be addressed urgently. For instance, the long-term stability of nanoparticles in real biological fluid environments, their sustained efficacy in tumor hypoxic areas, and the mechanisms of their distribution and clearance within the body still require further investigation. As Sancey et al. showed, Gd-based AGuIX nanoparticles accumulate and their clearance kinetics require deeper study [83]. Likewise, recent reviews underscore that in vivo biodistribution, metabolism and long-term fate of high-Z nanoparticles are among the major translational roadblocks [12,84]. Some studies suggest that cerium-oxide (CeO_2_) based nanoparticles may sustain ROS generation even under hypoxic tumour microenvironment conditions, thereby partially alleviating the impact of hypoxia on radiosensitisation. Although reports specifying activity at extremely low oxygen levels are still limited, the concept is increasingly supported by nanomaterial-based TME modulation research [85,86,87]. In mechanistic studies, immunohistochemical analyses have shown that tumour tissues treated with nanoparticle + radiotherapy may exhibit down-regulation of DNA-repair proteins (such as BRCA1, RAD51) and up-regulation of pro-apoptotic markers (such as Caspase-3, BAX). This is consistent with single-cell RNA sequencing data revealing suppression of repair pathways and enhancement of apoptotic signalling [88]. Although few studies report all these proteins in a single experiment specifically for lanthanide-based nanoparticle radiosensitisers, a growing body of evidence supports this overall pattern [14,89,90]. Based on these findings, optimizing the usage strategies of nanosensitizers, such as the injection-irradiation time interval, combined with DNA repair-inhibiting drugs, and enrichment to enhance tumor selectivity has become the current research focus.

It is worth noting that the pre-clinical studies also attach great importance to the construction of the theranostic platform. Using the MRI visibility already described, AGuIX allowed on-table re-contouring and dose-painting in pre-clinical models [63,91]. 

### 4.2. Clinical Trials

The clinical translation path of lanthanide-based nanosensitizers has progressed from the initial verification of feasibility and safety to the current stage of efficacy confirmation centered on randomized controlled trials. AGuIX first showed lesion-specific T1-MRI tracking; the subsequent two randomized Phase II studies, NANORAD-2 (WBRT + AGuIX) and NANOBRAINMETS (SRS/FSRT + AGuIX), were initiated and completed enrollment, aiming to evaluate whether the combination of AGuIX with standard radiotherapy could improve local control and intracranial remission endpoints (all achievable with quantifiable tracking in terms of equivalence and imaging), marking a crucial leap from imaging feasibility-safety controllability to efficacy validation [92,93].

In the primary central nervous system tumor, Nano-GBM (IB/II) included AGuIX in the standard program of radiotherapy + temozolomide [15]. The results of phase IB suggested that 100 mg kg^−1^ (four doses) was the recommended dose for phase II (RP2D), and no new dose-limiting toxicity related to AGuIX was observed. At the same time, Aguix was widely distributed and quantitatively enhanced in the tumor [93]. Combined with the optimization practice of VFA-T1 mapping and other sequences by the multi-center team, the correlation analysis between the tumor volume of nanoparticles and tumor response is forming a standardized process, which makes the closed loop of “image quantification-dosimetry coupling” more operable.

AGuIX’s clinical exploration also extends to the body tumors on MR-Linac platform. Nano-SMART (I/II) focuses on pancreatic cancer and central lung tumor, embeds the positive contrast characteristics of AG uIX’s MRI into the online adaptive SBRT workflow, and evaluates the real-world feasibility and early curative effect signal of tumor imaging-functional redrawing-dose increasing [94]. Correspondingly, NANOCOL (locally advanced cervical cancer, stage I) in the gynecological tumor direction quantifies the concentration of nanoparticles through patient MRI, and explores the potential of functional target redrawing at the node of synchronous radiotherapy and chemotherapy and intracavitary brachytherapy, which shows that gadolinium-based nano-drugs have room for extrapolation in the individualized radiotherapy process of imaging guidance. These attributes, namely high atomic number (Z), chemical stability, and facile surface chemistry, provide the material basis for their radiosensitizing function (mechanistic details are given in Section 5) [95].

It is worth noting that external comparison with the clinical evidence of non-lanthanide high Z nano-sensitizers is helpful to calibrate the expectation and evaluation framework. Taking hafnium oxide nanoparticles NBTXR3 as an example, the multicenter randomized phase 2–3 Act. In.Sarc test of soft tissue sarcoma showed that adding NBTXR3 on the basis of preoperative radiotherapy could significantly improve the complete remission rate of pathology (16% vs. 8%, *p* = 0.044) and improve the R0 margin rate. At present, the global stage ⅲ NANORAY-312 of head and neck squamous cell carcinoma is under way, further verifying its clinical value in the elderly/platinum-unsuitable population. Although NBTXR3 is not a lanthanide material, its high-Z nano radiotherapy sensitization mechanism is similar, and the experimental design and supervision path can be used for reference, which provides reference coordinates for the subsequent random and registration research of lanthanide platforms such as AGuIX [96,97].

### 4.3. Comparative Study with Other Nanomaterials

In order to establish the unique value of lanthanide nanomaterials in radiotherapy sensitization, in recent years, horizontal research often takes high-Z dose physical gain + biological synergy + integrated visualization of diagnosis and treatment as the evaluation framework, and compares it with gold nanoparticles (AuNP), iron oxide (Fe_3_O_4_), titanium dioxide (TiO_2_) and hafnium oxide (HfO_2_) and other systems in parallel [98,99,100]. Table 2 summarizes the representative lanthanide-based nanoradiosensitizer platforms and their current translational status, providing a benchmark for the following comparative discussion.

In the clinically relevant energy region and complex tumor microenvironment, lanthanide materials show unique combined advantages in visual sensitization, real-time dose carving and hypoxia adaptability by virtue of quantifiable MRI positive contrast development and red oxidation catalytic ability that can still be maintained under anoxic conditions [103,104]. These renal-clearable, T1-quantifiable features of AGuIX were exploited for MRI-guided dose-painting; This kind of clinical pathway has obtained prospective verification signals in people with brain metastases, and formed a technical extension from increasing dose to dose-painting, which is a characteristic advantage that most therapeutic nano-radiotherapy sensitizers do not have [64].

The comparison with gold nanoparticles best highlights the energy spectrum dependence differences. Au has a higher atomic number (Z = 79), and in the kV energy range where photoelectric effect predominates, it often shows a higher physical dose enhancement; however, under the commonly used MV photon beams, Compton scattering dominates, and the gap brought about by pure atomic numbers between materials is significantly reduced. The actual DEF is often more sensitive to particle size, concentration, energy spectrum of the incident beam, geometry, and biological distribution. Multiple comparative studies suggest that the DEF range of Au and high-Z systems like Gd under MV conditions is similar. The conclusion depends on the specific experimental setup rather than solely being determined by Z. This also explains why lanthanides do not have a significant disadvantage in MV radiotherapy scenarios, while their MRI visualization ability and quantifiable pharmacokinetics/distribution in the comprehensive evaluation actually hold an advantage [105,106].

When comparing iron oxides with transition metal oxides such as titanium dioxide, the limited ROS production brought about by the hypoxic microenvironment of tumors becomes a crucial dividing line. Hypoxia not only weakens the oxygen fixation effect of conventional radiotherapy, but also inhibits the oxidative stress process of various inorganic nanomaterials due to the enhanced intracellular reductivity. Therefore, Fe_3_O_4_ and TiO_2_ often exhibit a reduction in sensitization effect when exposed to strong hypoxia (≤2% O_2_). However, lanthanide oxides, relying on the reversible cycle of Ce^3+^/Ce^4+^ and the response to pH/oxygen tension, can maintain the activation of hydrogen peroxide and the generation of active substances such as ·OH in the hypoxic environment. Thus, they are more likely to retain or amplify the biological sensitization signals in low-oxygen-tolerant tumor models. It should be emphasized that this dual redox activity also brings a scenario-dependent promoting damage to tumors and possibly protecting normal tissues, suggesting that individualized optimization should be combined with imaging and pharmacokinetics in terms of dose and administration sequence [107,108].

Compared with the mature non-lanthanide reference path of hafnium oxide (NBTXR3), lanthanides share commonalities with high-Z physical weighting at the level, but present different routes in terms of integrated diagnostic and therapeutic capabilities and the integration of online adaptive workflows. The random evidence of NBTXR3 has verified the improvement of pathological remission rate and R0 resection rate in preoperative radiotherapy for soft tissue sarcoma, and is currently conducting a phase III extension in the head and neck squamous cell carcinoma scenario; while the lanthanide platform focuses on the core of imaging—tumor uptake quantification-dose sculpting for multiple tumor types, promoting the clinical paradigm of visualized sensitization [96,109,110]. The parallel development of the two provides a powerful reference for the external validity and evaluation benchmark of high-Z nano-sensitizers, and also indirectly confirms the decisive role of integration of workflow and imaging in determining clinical value beyond a single material parameter.

It is worth noting that the concept of composite and hybrid design is emerging as a cutting-edge approach to narrowing the gap between different systems. Integrating Gd with Au or other high-Z elements on the same platform not only enables the combination of the advantages of their electromagnetic interactions to increase the probability of ionization events, but also allows for multimodal imaging-treatment integration by leveraging Gd’s MRI positive contrast and Au’s optical/CT advantages. For instance, Kouri et al. discussed the potential of Gd-Au nanoparticles in cancer imaging therapy; Wang et al. reported the high sensitivity of Au/Gd_2_O_3_ complexes in imaging of nasopharyngeal cancer cells [101,102]. These alloying/nuclear-shell/point lattice matching strategies have shown stronger radiotherapy synergy in models such as breast cancer than with single materials, providing structural space for hypoxia alleviation, drug co-delivery, and immune activation; at the same time, systematic optimization of coatings and surface chemistry helps achieve a balance between delivery efficiency, aggregation/toxicity control, and physical dose enhancement, which is also a key link for the standardized clinical application of nanoradiotherapy sensitizers [111].

Overall, the lateral comparison does not simply provide a static ranking of who is absolutely stronger, but rather reveals a dynamic optimal solution closely related to the coupling degree of the energy spectrum, microenvironment, imaging science and workflow. Under the MV clinical range, the gap in physical DEF between the lanthanide series and Au and other high-Z systems has been narrowed, but the lanthanide series, relying on MRI visualization and hypoxia-adaptive catalysis, demonstrates unique advantages in visualization sensitization, online individualized dose management and low-oxygen tumor response; When compared with the random evidence formed by NBTXR3 as an external benchmark, it further highlights the importance of integrating imaging quantification—dosimetry—clinical workflow. Composite Gd-Au or Gd-Bi systems that incorporate biodegradable coatings are under active investigation to increase dose enhancement and simultaneously lower long-term retention [98,112]. Quantitative radiosensitization indicators for representative lanthanide and other high-Z nanosystems are summarized in Table 3.

Quantitative radiosensitization indices such as the Dose Enhancement Factor (DEF), Sensitization Enhancement Ratio (SER), or related metrics serve as more than mere numerical descriptors. They reflect the combined physical, chemical, and biological contributions of nanomaterials to radiation response. At the physical level, high-Z materials like hafnium oxide can significantly increase local energy deposition via photoelectric and Auger cascades, thereby elevating DNA damage yields; at the chemical level, they catalyze reactive oxygen species (ROS) formation, while at the biological level they enhance downstream damage signaling and cell kill. Importantly, these indices exhibit strong dependence on the radiation energy spectrum and experimental conditions. For example, gold nanoparticles show pronounced enhancement under low-energy (keV) X-rays but less so under clinical megavoltage beams, whereas HfO_2_-based systems retain a measurable enhancement across a broader energy range, consistent with their higher atomic number and physical cross-section advantages [117]. Clinically, such quantification is valuable for guiding nanoparticle selection and dose planning, as well as for interpreting comparative outcomes across different radiosensitizer platforms. In the case of NBTXR3 (hafnium oxide), both preclinical and early clinical evidence suggests that its quantitative enhancement correlates with improved tumor control and immune modulation when combined with radiotherapy.

## 5. Tumor Radiosensitization

With the rapid development of Ln-NPs, tumour radiosensitisation research has evolved from basic in vitro/in vivo validation towards image-guided, dose-optimised clinical translation. Building on the combination of high-Z physical weighting, ROS-mediated chemical amplification and MRI-visible theranostic design established in previous sections, this chapter focuses on how Ln-NPs enhance radiotherapy efficacy at the tumour level and how representative platforms have moved along the translational pathway.

### 5.1. Case Study of Success

A large number of studies and cases have demonstrated that lanthanide-based nanomaterials have proven their effectiveness and safety as radiosensitizers in various tumor models [23]. A representative preclinical success case comes from the bimetallic diagnosis and therapy nanoplatform AGuIX-Bi. In a mouse xenograft tumor model, AGuIX-Bi combined with irradiation significantly delayed tumor growth and achieved approximately 33% complete remission; the researchers attributed the improved efficacy to the high-Z interaction and increased secondary electron yield brought about by the synergy of gadolinium/bismuth, and the material itself retained MRI visibility to support precise delineation and dose concentration [91]. This combined approach directly showcases the closed-loop advantages of imaging sensitization within the same nanoscale framework.

In the field of composite nanostructures, the Gd_2_O_3_@BSA-Au core-shell nanoparticles provide empirical evidence for the gadolinium-gold synergy. This study completed in vivo and in vitro evaluations in a breast cancer mouse model. On the basis of X-ray irradiation, the composite particles significantly increased intracellular ROS levels and inhibited clone formation, and in vivo, they showed stronger tumor suppression and good systemic tolerance, with no major organ damage observed in pathology; based on this, the authors proposed a dual mechanism framework of physical dose enhancement + chemical amplification, providing a replicable template for subsequent optimization under more complex energy spectra and administration schedules [38].

What is more noteworthy is that the clinical feasibility of visual enhancement has been verified by the early studies of AGuIX. In multiple I/II/III phase cohort and protocol papers, AGuIX administered intravenously can form stable T1 enhancement within the tumor and is correlated with the response to radiotherapy, thereby supporting the functional re-contouring and dose-painting strategies based on MRI; The same research sequence also provided the recommended II phase dose (RP2D) and the safety of combined concurrent radiotherapy and chemotherapy, providing methodological and pharmacokinetic basis for cross-tumor expansion [92,118]. 

In the targeting and in vivo behavior characterization aspect, the ligand engineering and single-particle tracking techniques for Ln-NPs provide tools for mechanism verification and dose conversion. The review and original studies show that ligands such as RGD can enhance tumor integrin-mediated uptake and improve in vivo distribution [119]; at the same time, single-particle tracking (SPT) using upconversion or luminescence labeling provides a spatiotemporal resolution means for evaluating the dynamics, tumor entry path, and residence time of the particles in the tumor microenvironment, which helps to establish a closer quantitative correlation between imaging signals and effective doses [120,121]. Although differences in ligand density, particle size, and surface chemistry on different platforms may lead to quantitative variations, the causal chain of targeting—tumor entry—sensitization is becoming increasingly clear [122,123].

Furthermore, the redox-activatable lanthanide complexes are expanding the biological sensitization boundaries of Ln-NPs through the hypoxia response strategy. Some lanthanide complexes based on the DOTA framework can be designed as oxygen/reduction state-responsive probes or imaging-treatment components sensitive to ROS/RNS, achieving selective activation and continuous ROS production in the hypoxic environments of solid tumors, thereby forming a synergistic effect with the free radical mechanism of radiotherapy; Quantitative in vivo assessments of hypoxia-targeted lanthanide complexes have recently been reported [124], offering initial guidance for therapeutic window definition [125,126,127].

At the pre-clinical level, platforms such as AGuIX-Bi have demonstrated reproducible complete remission and significant growth inhibition; at the composite structure level, Gd_2_O_3_@BSA-Au has achieved synergy through both physical and chemical pathways; at the clinical process level, AGuIX has brought imaging-sensitization into an adaptive radiotherapy workflow centered on MRI. With the successive release of randomized control data and the more mature quantitative coupling of imaging—dose—efficacy, lanthanide-based nanomaterials are expected to form a visualized sensitization + individualized dose optimization robust paradigm in precise radiotherapy, and further generate verifiable synergistic effects with multimodal treatments such as photoacoustic therapy [128].

### 5.2. Enhancement of Treatment Effectiveness

Lanthanide-based nano-enhancers are gradually bringing the concept of visualized enhancement from the laboratory to the clinical pathway. As already described, intravenous AGuIX yields quantifiable T1-positive tumor enhancement with good tolerance. Taking brain metastases as an example, early studies such as NANO-RAD showed that AGuIX combined with whole-brain radiotherapy was verified in terms of safety and feasibility, and MRI can directly track the entry and retention of the nano-drug in the lesion; subsequently, the NANORAD-2 (NCT03818386) and NANOBRAINMETS randomized II phase trials advanced based on this, aiming to evaluate the true gain in intracranial remission and local control above standard radiotherapy. For primary central nervous system tumors, NANO-GBM 1b phase has reported the recommended II phase dose (100 mg kg^−1^, 4 times) and confirmed no new dose-limiting toxicity, while suggesting that AGuIX diffuses well in glioma lesions, providing pharmacokinetic and imaging-based evidence for imaging-dose coupling dose sculpting. Overall, the current clinical evidence supports the imaging-based individualized radiotherapy sensitization pathway, and the superiority of the efficacy endpoint still awaits the complete readout of ongoing randomized studies.

In the broader spectrum of solid tumors, AGuIX is being combined with the online adaptive SBRT technology of MR-Linac to explore the imaging quantification—functional re-contouring—dose escalation clinical advancement process [129]. Its core value does not lie in simply increasing the prescription dose, but in leveraging the intratumoral visual positioning and quantifiable distribution of nanomedicines to improve the dose and biological distribution match, thereby enhancing the conformity and consistency of tumor dose while potentially better protecting vital organs. Methodological and review studies have repeatedly emphasized that when nanomedicines provide contrast enhancement within the lesion + dynamic information, dose painting can move towards the image-dose closed-loop mode, and lay the foundation for the replicability of multi-center clinical workflows.

From a biological perspective, lanthanum oxides maintain the activation of hydrogen peroxide and the amplification of free radicals through the reversible cycle of Ce in the tumor-specific microenvironments of low oxygen and acidity. Thus, in the hypoxia-tolerance model, they can still retain or even enhance the sensitizing effect [130,131,132]. Early mechanism studies have shown a chain of radiotherapy-ROS accumulation-DNA damage enhancement-clonal formation inhibition in pancreatic cancer and lung cancer cell/autologous transplantation models; recent work has further combined nano-cerium with alleviating hypoxia and enhancing DNA damage response, suggesting its potential for radiotherapy synergy in non-small cell lung cancer and other refractory cancer types. It should be emphasized that these red-oxygen bidirectional nanoenzymes can not only promote oxidation and amplify free radical damage within tumors, but also may exhibit protective effects of eliminating oxidative stress under the physiological pH/oxygen tension of normal tissues. This requires combining imaging and pharmacokinetic quantification in terms of dose and administration sequence to maximize the ratio of tumor benefit/risk to normal tissue [133].

Compared with traditional chemotherapy-based sensitization strategies, high-Z nano-sensitizers are demonstrating the advantages of targeted loading in the target area and reduced burden in non-targeted areas in the therapeutic window. Currently, its III phase study in the head and neck squamous cell carcinoma scenario is underway [134]. These results validate the clinical feasibility of high-Z nano-sensitizers from an external control perspective, and also provide an evaluation framework and expected boundaries for the lanthanide platform. It is worth noting that AGuIX is currently still in the preclinical/early clinical stage, and its large-scale randomized controlled data have yet to be accumulated.

With the advancement of combined research on radiotherapy and immunotherapy, nano-sensitizers are expanding the discussion of therapeutic efficacy from pure local response to systemic immune response. Preliminary animal studies have shown that these nanoparticles combined with radiotherapy can enhance radio-induced immunogenic cell death (ICD), promote dendritic cell activation and effector T cell infiltration. Further, when this approach is combined with PD-1/PD-L1 immune checkpoint inhibition, although direct data is limited, existing literature supports the possibility of inducing distant (abscopal) effects. This direction is consistent with the overall trend of enhancing cross-sensitization of radiotherapy and immunotherapy with nanotechnology. It also suggests that in future clinical designs, remote lesion control and immune biomarkers should be considered as combined endpoint evaluation indicators [135,136,137].

### 5.3. Reduction of Side Effects

The ideal radiotherapy sensitizing agent should not only enhance local control, but also reduce treatment-related toxicity and improve the quality of life throughout the entire treatment course. The advantages of lanthanide nanomaterials in this regard come from two complementary pathways: one is selective enrichment and visual delivery, allowing target area loading and non-target area reduction to truly take effect; the other is context-dependent redox regulation, which ensures sensitization within the tumor while presenting potential protective effects on normal tissues. The former achieves more precise delivery of energy to the lesion through an imaging-dosimetry closed loop, while the latter provides a new biological anchor for toxicity management by regulating oxidative stress through the material’s inherent enzymatic activity. Early clinical studies have accumulated relatively consistent safety and tolerance signals, providing a basis for advancing “sensitization + reduction of toxicity” from concept to process [77].

Firstly, the enrichment advantage resulting from the combination of passive EPR and active targeting is the basis for reducing the exposure of adjacent tissues and side effects. AGuIX’s sub-5 nm size exploits EPR, permits renal clearance and enables MRI-based dose quantification. Relevant studies have successively shown from animals to clinical trials. AGuIX forms a stable T1 enhancement in brain metastases and can be quantified using VFA-T1 mapping; these visible nanomedicines enable radiotherapy plans to be re-sketched functionally and dose-punched based on the actual drug distribution, and are expected to further reduce the dose to organs at risk while ensuring tumor coverage [138].

Secondly, image-guided online adaptive radiotherapy provides a new workflow approach for toxicity management. The development of MRI-Linac has made same-machine imaging—same-machine irradiation a reality; when nanoparticles themselves are MRI positive contrast enhancers, clinicians can confirm the tumor entry and time phase during the treatment session, and accordingly adjust the dose allocation and field shape, reducing unnecessary exposure of normal tissues at the process level [139,140]. This concept has been systematically elaborated in the preclinical and clinical methodology studies of AGuIX, using MRI to quantify tumor entry, implementing dose-painting or moderate dose escalation based on this, while improving the conformality of the target dose, theoretically reducing the cumulative burden of organ-risk volumes, and thereby translating into toxicity benefits in skin/lung/brain and other areas.

Furthermore, the selective redox regulation of lanthanum-oxide provides a biological basis for the potential attenuation of normal tissues [141,142,143]. Nanomaterials such as CeO_2_ possess a reversible Ce cycle, which can exhibit free radical scavenging and antioxidant properties in normal tissue environments with lower oxidation pressure and a pH closer to physiological conditions, thereby alleviating radiation-induced damage to the epidermis, glands, and mucous membranes. In the tumor microenvironment (with low oxygen and higher reducibility), they are more likely to promote peroxide activation and ROS amplification, thereby synergistically killing with radiation. 

Finally, safety and pharmacokinetic evidence establish the bottom line and boundaries for controlling side effects. These safety findings were confirmed at 100 mg kg^−1^. The main elimination route was rapid clearance through the kidneys; the spectrum of adverse events was mainly controlled reactions related to radiotherapy, and had a traceable time-dose relationship. The quantitative-intratumor-dose-reaction association study that accompanies it is being refined, providing a path for directly translating imaging parameters into toxicity and efficacy predictions in the future.

## 6. Discussion 

Although we have summarized the physicochemical foundations, radiosensitization mechanisms, and experimental paradigms of lanthanide-based nanomaterials, a unified interpretation reveals that their radiosensitizing effect arises from the coupling of physical, chemical, and biological processes rather than from any single dominant pathway. High atomic number–mediated secondary electron emission provides the initial physical amplification, which is subsequently magnified by catalytic reactive oxygen species generation and interference with DNA damage response pathways. Emerging evidence further suggests that these effects may extend beyond direct tumor cell killing to influence immune-related signaling; however, the relative contribution of each component remains context-dependent. Importantly, under clinically relevant megavoltage irradiation, the magnitude of physical dose enhancement alone appears modest, indicating that chemical redox activity and biological modulation are likely indispensable contributors to the overall radiosensitization observed in vivo. This integrated view explains why variations in nanoparticle composition, size, surface chemistry, and tumor microenvironment frequently lead to heterogeneous experimental outcomes across studies. 

From a translational perspective, the accumulated preclinical literature demonstrates reproducible radiosensitization across multiple tumor models, particularly when lanthanide nanomaterials are combined with image-guided delivery or multimodal treatment strategies. Nevertheless, the strength of evidence is uneven, as many studies rely on distinct irradiation conditions, biological end points, and nanoparticle characterization standards, limiting direct cross-comparison. Early-phase clinical investigations, exemplified by gadolinium-based platforms such as AGuIX, provide proof-of-concept for safety and tumor-localized MRI contrast during radiotherapy. However, objective evidence for improved clinical outcomes remains preliminary, as randomized efficacy end points and standardized dose–response correlations are still lacking. In this regard, the current clinical trajectory of lanthanide-based radiosensitizers closely parallels earlier experiences with other high-Z nanoparticles, underscoring that technical feasibility does not automatically translate into therapeutic superiority over contemporary radiotherapy techniques.

## 7. Limitations

This chapter will focus on three major aspects: toxicity and biocompatibility, delivery and targeting, and regulation and ethics, to discuss the existing problems and solutions. These challenges need to be addressed urgently to ensure that lanthanide-based nanomaterials sensitizers can fully exert their therapeutic effects while ensuring patient safety.

### 7.1. Toxicity and Biocompatibility

Safety has always been the threshold that new nano-medical materials must overcome. In vivo fate and potential organ accumulation strongly depend on particle physicochemistry. PEG- or silica-coated LnNPs frequently demonstrate negligible acute cytotoxicity at low doses, whereas uncoated cores and higher exposures can provoke oxidative stress and inflammatory responses [144,145]. Quantitative analyses using techniques such as ICP-MS and SPECT/CT show preferential accumulation in liver and, for some radiolanthanide forms, in bone epiphyseal regions, consistent with prolonged organ deposition patterns.

The potential toxicity and biocompatibility issues of lanthanide nano-materials in the body require comprehensive assessment and overcoming. Taking the gadolinium-based system as an example, the gadolinium element itself is relatively safe when used as an MRI contrast agent in the chelated state, but when applied in the form of nanoparticles for treatment, its long retention and accumulation effects are of concern. Especially for patients with renal dysfunction, a large dose of gadolinium has the risk of inducing renal fibrosis [146]. Therefore, optimizing the formulation to make the nanoparticles removable becomes crucial. Although AGuIX has good renal clearance properties, clinical observations have found that the clearance rates vary greatly among different patients. Additionally, the size, shape, and surface chemistry of nanoparticles will affect their toxicity profile [147]. Generally, smaller particle sizes are more likely to penetrate the biological barrier but may remain in the body for a longer time, and particles with sharp morphology or positive charge are more likely to interact with the cell membrane and cause cytotoxicity [148,149]. Preclinical biodistribution and toxicity studies of lanthanide-based nanoparticles indicate that particle size, surface chemistry, and coating strongly determine in vivo fate and organ retention. Notably, ultrasmall Ln-NPs are often rapidly eliminated via renal pathways, whereas larger particles accumulate in organs associated with the mononuclear phagocyte system such as liver and spleen, with additional evidence of persistent signal in bone tissue beyond 30–60 days post-administration [150,151]. Mechanistic studies have also reported oxidative stress and ion homeostasis disruptions in central nervous system tissues at high exposure levels. However, comprehensive chronic toxicity studies integrating functional end points across CNS, hepatic, and skeletal systems remain scarce. These preclinical results are valuable for safety margin estimation and protocol development for future chronic toxicity evaluations but cannot alone substitute for formal long-term toxicological assessments required before clinical translation.

The safety of bimetallic systems is even more complex. Nanoparticles like Gd@Bi introduce a second metal, and their toxicity depends on the individual effects of each component as well as their interaction. Although bismuth enhances the sensitization effect, its metabolic pathway and potential long-term effects in the body remain unclear [152]. The stability and toxicity of nanoparticles will also change with different component ratios. This requires evaluating the dose-effect and dose-toxicity relationships of different ratios one by one to find a combination that is both efficient and safe.

The complexity of the tumor microenvironment also affects the biocompatibility and degradation behavior of nanoparticles [153,154,155]. Solid tumor tissues are often acidic, hypoxic, and have abnormal blood vessels, which may cause nanoparticles to undergo degradation release or aggregation outside of the expected location. For example, some nanoparticles may aggregate or precipitate in the acidic pH of the tumor, thereby increasing local toxicity. Researchers attempt to modify the surface of the particles with polyethylene glycol (PEG) or amphoteric ion polymers to reduce plasma protein adsorption and enhance the stability of the particles in circulation [156]. However, such modifications may not be effective in the tumor environment; some reports indicate that certain lanthanide nanoparticles will slowly form small aggregates in the blood and be recognized and cleared by macrophages, resulting in excessive accumulation in the liver and spleen [157]. Therefore, we need to gain a deeper understanding of the interaction mechanism between lanthanide nanoparticles and biological macromolecules, including how to prevent their aggregation in plasma and how to maintain good dispersion in the tumor matrix. These knowledge will guide us in designing safer and more controllable nanoparticles.

To address these issues, a multi-pronged strategy is being explored. The first step is to develop degradable lanthanide complexes or carriers, so that the particles can be metabolically cleared after completing the sensitization task. For example, lanthanide elements can be encapsulated in biodegradable polymers or liposomes, and their degradation release can be triggered by enzymes or pH in the body. This ensures both short-term efficacy and reduces long-term burden. Secondly, fine surface engineering can improve biocompatibility: by introducing tumor-specific ligands on the outer layer of the particles, not only is the targeting ability enhanced, but also the retention time in normal tissues is reduced, as the ligand-mediated tumor enrichment is completed, the unbound particles are more likely to be excreted. Finally, a strict in vitro and in vivo toxicity screening system must be established. The toxicity of nanoparticles may not be obvious in cell experiments, but it shows cumulative effects in animals and even humans, so it requires months or even longer follow-up observations and multi-index evaluations, such as inflammatory factors, immune responses, and tissue pathology. Standardized characterization and testing are also indispensable: currently, different studies have different methods for characterizing nanoparticles and toxicological tests, making it difficult to compare data crosswise. Uniform standards should be established, such as requiring the reporting of key parameters like particle size distribution, zeta potential, and stability in different physiological media, and using standard cell lines and animal models to assess toxicity, so that the results of different teams can be comparable.

Overall, although the challenges of toxicity and biocompatibility are severe, they are not insurmountable. With the close collaboration between materials science and biomedical science, we are gradually understanding and mastering how to minimize toxic side effects while retaining the sensitizing effect. For example, by optimising particle size and surface properties, lanthanide nanomaterials can enhance radiotherapy while being rapidly cleared from the body; in parallel, biomimetic surface coatings that disguise nanoparticles from immune recognition are being explored to prolong circulation time [158].

### 7.2. Delivery and Targeting

Achieving efficient and precise in-body delivery is another key factor determining the efficacy of lanthanide-based nanosensitizers. The traditional passive targeting relies on the enhanced permeability and retention effect (EPR effect) of tumors, where nanoparticles enter the tumor through abnormally dilated blood vessels and remain in the interstitial space [159]. However, the EPR effect varies greatly among different tumor types and individuals, being influenced by vascular permeability, interstitial pressure, and lymphatic drainage efficiency, resulting in an often uneven distribution of nanoparticles in tumor tissues. This spatial heterogeneity leads to the formation of “cold zones” in tumors, where some cells may not receive sufficient doses. Therefore, the active targeting strategy has emerged, which significantly increases the uptake of nanoparticles at the lesion site by modifying specific ligands to recognize and bind to tumor cell receptors. However, active targeting also faces the protein charge problem. Plasma proteins adsorb after modification, masking the ligand recognition sites and weakening the ability to bind to receptors. Additionally, the in-body enzymatic degradation may break peptide ligand bonds, further affecting the targeting efficiency.

In terms of tumor internal diffusion, the physical properties of the particles are also crucial. The dense extracellular matrix and high interstitial pressure of solid tumors hinder the deep penetration of most nanoparticles. Experimental and simulation studies have shown that nanoparticles with a particle size of 10–30 nm often can balance both permeability and retention, achieving a more uniform distribution in tumor tissues [160]. Fine-tuning of surface charge can also improve diffusion characteristics, slightly negatively charged particles are stable in the blood and less prone to non-specific adsorption, while particles with overly strong positive charge are cleared in the early circulation. The biological barriers are another challenge in delivery. For the treatment of brain tumors, focused ultrasound (FUS) assisted microbubble technology can temporarily open the BBB, allowing lanthanide nanoparticles to cross the barrier and achieve local aggregation [161]; for the problem of RES clearance, PEG modification can prolong the circulation time in the blood, but it also weakens cellular uptake, creating a contradiction between invisible coating and targeting. The recently emerging biomimetic coating strategy, such as encapsulating nanoparticles with red blood cell membranes or tumor cell membranes, enabling immune evasion and enhancing stability in the body, has emerged as a promising improvement direction [162].

The trade-off between delivery efficiency and targeting accuracy remains a core issue in design. Nanoparticles modified with large molecule ligands can highly specifically guide them to the surface of tumor cells, binding to specific receptors, but antibody molecules are large and structurally rigid, often limiting the diffusion of nanoparticles to the deep parts of tumor tissues. Moreover, the ability of antibodies to penetrate tumors is also limited, with a common phenomenon of concentrating only near tumor blood vessels and being unable to reach distant tumor cells. In contrast, small molecule ligands or peptides have better permeability due to their small size and good flexibility, being able to more evenly spread throughout the tumor tissue, but their specificity and affinity are relatively lower than antibodies, and their half-life in the body is short [163]. Some studies have compared the differences between peptide and antibody as targeting ligands, finding that peptides are more efficient in tissue penetration and endocytosis, while antibodies excel in binding stability and immune activation [164]. In response to this, some scholars propose to select appropriate targeting strategies based on tumor type and location, achieving personalized optimization [165], for brain tumors, more emphasis is placed on penetration and widespread distribution, and small-sized RGD-modified nanoparticles may be superior to large antibody-modified nanoparticles; for tumors with abundant blood supply and clear specific antigens, antibody-mediated targeting may be more effective. In the future, radiogenomics and imaging genomics can be utilized to analyze the vascular characteristics, receptor expression, and microenvironment status of patients’ tumors, thereby enabling the selection of the most optimal nano-sensitizers and delivery schemes for each patient [166]. In fact, there is a viewpoint suggesting that stratification based on the specific characteristics of patients and tumors should be carried out, and those tumors with weak EPR effects should be selected and given additional targeted/penetration-enhancing measures, while tumors with strong EPR effects can adopt a conventional passive targeting-based strategy.

Furthermore, new technologies have opened up pathways to improve delivery efficiency. Computational fluid dynamics (CFD) simulations and multi-compartment pharmacokinetic modeling have been used to study the transport process of nanoparticles in tumor blood vessels and tissues, helping to optimize the particle size and dosing regimen. For instance, a mathematical model simulated the distribution of nanoparticles of different sizes in solid tumors, and the results suggested that particles of approximately 100 nm remained in the extracellular space outside the blood vessels the longest, while particles of around 20 nm had the best penetration depth into the tumor, and the ideal size should be comprehensively considered [167]. Another example is the multi-compartment model mentioned above, which divides tumor tissues into vascular, extracellular space, and intracellular regions, and conducts dynamic simulations of AGuIX accumulation and clearance in tumors, enabling more accurate prediction of the effects of different sequential administrations. At the same time, machine learning is extracting patterns from a large number of animal experiments and clinical data to predict which nano-material designs are most effective for specific tumors [168]. Existing studies have trained deep neural network models using data on the delivery efficiency of dozens of nanocarriers in different tumors, successfully predicting the delivery performance of various new nanocarriers in specific tumors and identifying the key parameters affecting delivery. These intelligent algorithms can significantly accelerate the development of nanomedicines, shifting the design process from being driven by experience to being data-driven [169,170].

Overall, lanthanide-based nanomaterials still face multiple challenges in safety, delivery, and regulation, but these issues are gradually being resolved with interdisciplinary collaboration and technological innovation. Through the comprehensive optimization of degradable structure design, targeted functionalization, and individualized delivery strategies, lanthanide-based nano-sensitizers are expected to achieve the dual goals of efficient sensitization and low-toxic delivery in radiotherapy, paving the way for the clinical application of precise radiotherapy [171].

### 7.3. Regulatory and Ethical Issues

As lanthanide-based nanosensitizers gradually move towards clinical application, regulatory and ethical issues are increasingly prominent. For the novel radiopharmaceutical formulations described in this review, the structure of future quality standards should integrate both classical pharmaceutical quality controls and radiochemistry-specific criteria. In addition to conventional assessments of chemical and physical purity, formulation stability, and sterility, radiopharmaceutical products require explicit measurement and control of radiochemical purity (RCP), radionuclidic purity, and specific activity expressed in appropriate units and referenced to a defined activity reference time. International guidelines and pharmacopeial monographs establish RCP and radionuclidic purity limits for diagnostic and therapeutic radiopharmaceuticals, and emphasize the necessity of reliable radiolabeling and stability testing prior to clinical use. Moreover, good manufacturing practice (GMP) principles and quality assurance systems need to encompass specialized radiopharmacy requirements, including process validation, equipment qualification, and real-time activity measurement and documentation. Taken together, these elements form a comprehensive quality standard framework that ensures consistent product quality, safe handling, and clinical effectiveness of future radiopharmaceutical drugs.

At the regulatory level, currently, the drug regulatory agencies have not yet formulated specific guidelines for lanthanide-based nanomedicines. The current regulations mostly follow the frameworks of previous small molecule drugs or ordinary nanomedicines. However, lanthanide-based nanosensitizers have both diagnostic and therapeutic functions, and their mechanism of action is complex, which may require new interdisciplinary assessment standards. Traditional toxicological evaluation needs to incorporate considerations of the safety of long-acting imaging contrast agents and the assessment of potential environmental residual lanthanide elements. The lack of a unified standard leads to an unclear regulatory path, and researchers face uncertainty when applying for clinical trials or obtaining marketing authorization.

In terms of ethical considerations, gadolinium and other lanthanide elements have raised concerns due to the delayed side effects reported in previous contrast agents. Some patients have doubts about nanomaterials, fearing their long-term retention and unknown consequences. This requires us to weigh the benefits and risks ethically: for patients with potential significant benefits in the advanced stage, perhaps they can tolerate certain unknown risks, while for early-stage patients, more caution is needed. Additionally, lanthanide-based nanoparticles as integrated diagnostic and therapeutic reagents may involve patient data privacy, so it is necessary to fully inform and protect patient rights during informed consent. Public perception of nanomaterials is also influenced by past events, such as the failure lessons of certain nanomedicines and news about heavy metal pollution in the environment. Therefore, researchers and clinical doctors have the responsibility to openly and transparently communicate the safety evidence of lanthanide-based nanosensitizers, clarify misunderstandings, and build confidence.

Comparisons of regulations show that lanthanide-based nanomedicines have unique challenges compared to other nanomedicines. For example, as the first of this type of platform, AGuIX requires the regulatory authorities to consider whether its imaging monitoring function needs additional review; for instance, different countries have different regulations for radioactive substances or heavy metal drugs, requiring international coordination. The positive aspect is that the clinical advancement of AGuIX has accumulated cases for regulation, and similar products can refer to its path. Additionally, dynamic monitoring trial designs are new in traditional approval processes and require regulatory innovation to accommodate. There are already calls internationally to establish cross-border collaboration to unify nanomedicine standards and have multidisciplinary experts jointly develop guidelines to ensure neither innovation is suppressed nor safety is compromised.

Ethically, there are also issues related to environmental and social impacts. The large-scale use of lanthanide-based nanomedicines may lead to environmental residues, how to recycle and dispose of them, and the impact of cost-effectiveness on the fairness of patients in different regions, are all issues that need to be considered. It is necessary to assess the long-term effects while collaborating with ethicists, policymakers, and other stakeholders during the advancement of technology. In summary, in the process of lanthanide-based nanosensitizers moving towards clinical widespread adoption, we need a complete regulatory framework to guide product development and approval, and uphold patient-centered ethical principles, actively communicating with the public. Only in this way can we ensure that this new technology is safe, effective, and accepted by society, truly benefiting the majority of patients.

## 8. Outlook

Looking ahead, lanthanide-based nanomaterials will continue to advance along multiple cutting-edge paths in the field of tumor radiotherapy sensitization. With the overall progress of nanomedicine, we anticipate exciting breakthroughs in areas such as synthesis techniques, characterization methods, multi-therapy integration, and personalized medicin.

### 8.1. Innovative Synthesis Technology

Precise and efficient synthesis techniques are the cornerstone for achieving breakthroughs in the next generation of lanthanide-based nanosensitizers [172,173]. One of the future trends is to achieve atomic-level control and scalable amplification of nanoparticle synthesis through microfluidic technology. Microfluidic reactors can significantly enhance the precision of reaction conditions, such as temperature gradients and uniformity of reaction time, by mixing reagents in microscale channels. This method has successfully produced monodisperse gadolinium oxide nanoparticles and can regulate their lattice defect density, thereby directly influencing radiation absorption and energy conversion efficiency. What is even more remarkable is that the microfluidic process saves approximately 40% of energy consumption, making it more environmentally friendly compared to traditional solvothermal methods [174]. It is foreseeable that with the development of equipment and processes, microfluidics will become a powerful tool for the industrial production of high-quality nanomedicines.

Green synthesis is also an important direction for the future [175,176]. Besides the previously mentioned biological reducing agents, other green methods such as photochemical synthesis, electrochemical deposition, and template self-assembly are also worthy of exploration. The goal is to obtain high-performance nanoparticles while reducing toxic reagents and by-products. For example, preparing lanthanide nanoparticles by reducing metal salts with plant extracts not only has an environmentally friendly process but also often has bio-molecular modifications on the product surface, making them more biologically active [177]. Continuous flow processes combined with online monitoring are also effective solutions for achieving scalability, ensuring the consistency of each batch of products. It is believed that in the context of increasingly strict environmental requirements, green synthesis will receive more attention and make significant progress.

Another frontier is intelligent synthesis, which is a synthesis strategy that integrates advanced characterization and automated optimization [178,179,180]. For example, using in-situ X-ray absorption spectroscopy (XAS) to monitor the evolution of the valence state of lanthanum during nanoparticle generation, adjusting the addition rate or temperature of precursors based on real-time data, and thus modulating the properties of the products. This manufacturing while observing method can ensure that nanoparticles continue to grow towards the ideal state. Similarly, single-particle tracking technology can be used to optimize the surface functionalization process: by observing the changes in the fluorescence intensity of individual nanoparticles, one can determine the extent of ligand coverage and guide the reaction when to terminate. With the introduction of machine learning, we can even achieve adaptive synthesis—AI can adjust experimental conditions in real time based on online characterization results and automatically optimize synthesis parameters. This will greatly improve research efficiency and product quality.

Overall, future synthesis techniques will advance in four dimensions: precision, greenness, intelligence, and scalability. Whether the ability to produce large quantities while maintaining consistent performance will determine whether lanthanide-based nanosensitizers can be widely used in clinical settings. The continuous emergence of innovative synthesis techniques indicates that this goal will eventually be achieved, providing a continuous supply of high-quality nanomedicines for clinical use.

### 8.2. New Characterization Techniques

The advancement of characterization techniques has provided us with powerful tools for in-depth understanding and improvement of lanthanide nanomaterials. In recent years, with the introduction of ultra-high-resolution imaging methods such as cryo-electron microscopy (cryo-EM) into the field of nanomaterials, we have been able to observe the structure and behavior of nanoparticles with unprecedented precision [181,182,183]. Specifically, for lanthanide nanoparticles, cryo-EM is expected to directly analyze the arrangement configuration of surface ligands, the assembly state between particles, and the morphological changes in the real environment within cells [184,185,186]. For example, although current research using lanthanide nanoparticles as sensitizers is mostly focused on functional validation, by observing their localization in cells, contact with cell membrane, and physical binding with biological macromolecules using ultra-high-resolution electron microscopy, it will provide key clues for explaining the differences between in vivo behavior and in vitro models [187,188]. At the same time, the application of light source technologies such as synchrotron radiation X-ray absorption spectroscopy (XAS) is advancing, and capturing the valence state changes of lanthanum elements during irradiation (such as the transformation from Ce^3+^ to Ce^4+^) at the femtosecond to picosecond scale will help link the rate of free radical generation with the dynamic state of lanthanide elements, which is crucial for understanding the quantitative relationship between the redox cycle mechanism and radiotherapy sensitization.

On the other hand, multimodal joint characterization is becoming a trend, which is promoting the possibility of coupling location—structure—function studies [189,190]. Combining fluorescence microscopy techniques with electron microscopy-related imaging (CLEM, correlative light and electron microscopy) allows for first locating nanoparticles in cells through fluorescence labeling, and then switching to electron microscopy to observe their ultrastructural environment. This enables observing whether the nanoparticles are near the nucleus, beside the mitochondria, or have escaped from lysosomes into the cytoplasm [191,192,193]. Such techniques are particularly important in the study of lanthanide sensitizers, as their design often emphasizes nuclear localization or lysosome escape strategies. Mass spectrometry imaging (MSI) can further quantitatively map the spatial distribution of lanthanum elements at the single-cell or tissue level, and by sampling at different time points, it can depict the entire process from drug administration, distribution, action to clearance of nanoparticles. This panoramic time-space distribution map will guide us in optimizing doses, adjusting dosing frequencies, and reducing non-targeted exposure to normal tissues.

Furthermore, time-resolved techniques are also regarded as the focus of the next generation of research. During radiotherapy sensitization, the physical and chemical processes triggered by nanoparticles may be completed within an extremely short time scale, such as secondary electron release, free radical generation, DNA break initiation [194,195,196]. By using single-molecule fluorescence lifetime imaging, transient absorption spectroscopy or femtosecond-picoscale X-ray pump-probe techniques, we have the potential to monitor the state changes of nanoparticles at the moment of irradiation or immediately after irradiation in real time. This will reveal key questions such as whether the ROS induced by nanoparticles bursts at the moment of irradiation or is continuously generated, and whether its effect on DNA is mainly during the irradiation stage or continues after the irradiation. The data obtained through these techniques will provide a basis for us to establish a quantitative model of nanoparticle action—time kinetics—biological effect, thereby considering parameters such as the duration of action, the optimal irradiation time window, and the rate of particle clearance when designing sensitizers.

With these emerging characterization techniques, we are gradually solving the multi-dimensional puzzles of nanosensitizers in structure, behavior and mechanism of action. This understanding, in turn, will guide the rational design of materials, forming a characterization-design-recharacterization closed-loop improvement mode. In other words, by more precisely monitoring the behavior of particles in the body, optimizing their size, morphology, surface ligands, dosing sequence and dose, we are getting closer and closer to the ideal sensitizer. It can be predicted that when technologies such as single-particle tracking, nanosecond-level reaction imaging, in situ elemental valence state monitoring, etc. become more mature in the future, the performance of lanthanide nanosensitizers will be further refined, and the reliability of their actual clinical translation will also be significantly enhanced.

### 8.3. Combination with Other Therapies

Facing the multi-modal comprehensive treatment landscape of radiotherapy + X, the most valuable aspect of lanthanide-based nanomaterials lies in their ability to serve as both physical sensitizers and as a visual carrier and multi-drug platform for diagnosis and treatment integration, thereby weaving chemotherapy, immunotherapy, and photodynamic/photothermal therapy into an image-controllable closed-loop process. Within this framework, the visual information obtained through imaging is no longer merely auxiliary assessment, but directly enters the decision-making loop. Based on the T1 positive contrast imaging and quantitative tumor detection using gadolinium-based nanomaterial platforms, dose shaping and timing selection have data anchors, and have been systematically explained and verified in the MRI-Linac workflow and clinical studies, laying the methodological foundation for individualized multimodal combination therapy on an as-needed basis [197,198].

The combination of chemotherapy is the most direct and easiest to engineer path. Previous studies have shown that the synergistic use of platinum-containing drugs with high-Z nanosystems can enhance radiation-induced DNA breaks and improve cell killing efficiency [199,200,201]; this idea has been repeatedly proven in the combination of gold nanoparticles and cisplatin, suggesting that there is a stacking space between chemotherapy sensitization—nanosystem physical weighting—radiotherapy [202,203,204]. Transferred to the lanthanide platform, through controlled bonding or encapsulation on the surface of gadolinium-based or lanthanide-doped carriers, it is possible to maintain or even amplify the anti-tumor effect while reducing the systemic dosage, and achieve in-situ monitoring of the delivery and release process through MRI visualization, providing a basis for dose allocation and radiotherapy rhythmology.

The synergy with immunotherapy shifts local killing to systemic response. Multiple cutting-edge studies have indicated that gadolinium-based nanoparticles combined with radiotherapy can enhance immunogenic cell death and reshape the tumor immune microenvironment [205,206,207]; when combined with PD-1/PD-L1 inhibitors, not only does local lesion control improve, but also a remote effect amplification can be observed in bilateral or metastasis models, even in low-dose irradiation scenarios [208,209,210]. This evidence chain is becoming increasingly complete from mechanism to animal models, providing reproducible biological and therapeutic support for the nanosensitization—radiotherapy—immunotherapy triple path, and providing a basis for clinical trials to include immunocomplex endpoints (such as distant lesion response and immune biomarkers).

The combination of photodynamic/photothermal therapy and radiotherapy provides a natural arena for lanthanide-based materials [211,212,213,214]. Lanthanide-doped nanoparticles can achieve upconversion luminescence or photothermal conversion under near-infrared excitation, and can also be driven by radiation-induced luminescence to activate photosensitizers under X-ray irradiation, thus making light treatment of deep lesions possible; when combined with radiotherapy, the thermal stress and oxidative assault can form a co-directional synergy with radiation damage, and further improve the sensitivity to radiation by improving tumor oxygenation. Recent reviews and new material reports on lanthanide-doped photothermal/upper-conversion systems have confirmed the stable optical response achievable at NIR-detectable tissue depths, and demonstrated the feasibility of significantly enhancing photothermal efficiency and therapeutic synergy in composite nanosystems, laying the material and energy foundation for light-radio combination in tumors that are difficult to reach [215,216,217].

For multimodal therapy to truly take root, it is necessary to integrate the visualized nanoscale platform into the digital foundation of adaptive radiotherapy [218]. The rise of MRI-Linac and online adaptive radiotherapy (MRgART) has made the closed loop of imaging at the time–planning at the time–irradiation at the time a clinical reality; in this scenario, the tumor uptake signals of nanomedicines such as AGuIX can be quantified on the same day and used to guide dose escalation, avoid critical organs, and optimize the fractionation rhythm. At the same time, the rapid progress of artificial intelligence in organ segmentation, motion field estimation, dose accumulation, and plan re-optimization is transforming the dynamic coupling of image—dose—biological distribution into executable workflows, enabling real-time updates of multi-modal decisions such as when to administer the nanomedicine, when to adjust the dosage, and whether to combine immunotherapy/chemotherapy within regulatory boundaries. With the accumulation of evidence-based evidence for MR-Linac and AI-ART, this combination therapy using lanthanide nanomaterials as the visual anchor point has a greater chance of achieving replicable, multicenter consistent efficacy and safety benefits [219,220,221].

In conclusion, the role of lanthanide nanomaterials is shifting from the enhancer supporting role in radiotherapy to the connector and catalyst for multi-therapy synergy. They integrate drug chemistry, immune regulation, and optical energetics onto the imaging-visualized sensitization base, and then use MRI-Linac and AI-ART to achieve individualized, online, and verifiable strategy planning. With the further enrichment of preclinical and clinical evidence, this path is expected to incorporate local control, distant immune response, and toxicity constraints into the same framework, maximizing tumor clearance while minimizing systemic burden, and promoting stubborn tumor treatment from multi-line warfare to true integration warfare.

### 8.4. Personalized Medical Strategies

Entering the era of precision medicine, individual differences have become the key variable determining the success or failure of radiotherapy [222,223]. The integrated diagnostic and therapeutic properties of lanthanide nanomaterials make them naturally suitable for embedding in an evidence-based individualized approach: imaging visualization provides quantitative evidence of the tumor penetration volume and timing, materials science can rapidly iterate in terms of particle size, surface chemistry and functional ligands, while radiomics/genomics and AI will converge these evidences into executable decisions. This closed loop is not a generalized a nanomedicine drug is applicable to everyone, but rather treatment as needed for specific patients, specific tumor niches and specific irradiation schemes. In this scenario, gadolinium-based platforms have proven in early clinical trials the feasibility of visible tumor penetration and clear renal clearance, and based on this, multiple studies aimed at random control verification have been carried out, providing a clinical-level workflow model for individualized nanosensitizers [15].

The first step of individualization is to identify who is most likely to benefit. Radiogenomics is mapping the molecular landscape that affects radiosensitivity: abnormalities in pathways related to DNA damage response (DDR) and homologous recombination deficiency (HRD) often indicate greater sensitivity to high Z sensitization and free radical amplification mechanisms [224,225]; models linking tumor gene expression to radiotherapy outcomes are being driven by high-throughput genomics and deep learning, moving towards bedside-applicable predictive tools. Meanwhile, modern oncology has incorporated companion diagnostics into the main line of drug development, moving from identifying target sites to identifying the target patient population. The nano-diagnosis and treatment platform can also follow this strategy. Before treatment, it can stratify patients based on molecular/receptor profiles and quantitative imaging thresholds, and then decide whether to administer the drug, which type of nano-sensitizing agent to use, and how to combine it with radiotherapy [226,227,228,229].

In vitro functional validation provides a second layer of support for selecting the right person and using the right drug. Patient-derived organoids (PDO) can rapidly replay the actual responses of a patient’s tumor to different energy spectra, segmentation schemes, and nano-formulations while preserving key microenvironmental elements. The complex organoid model with combined immune/matrix co-culture is expanding this validation from cytotoxicity to the level of immune-radiotherapy linkage. Through PDO as an intermediary, multiple candidate lanthanide nanoplatforms can be parallelly screened, and the optimal patient-nanoparticle-irradiation combination can be matched, feeding back to clinical protocol design and dosimetry settings [230,231].

Customized preparation enables the selected best formulation to be rapidly transformed into an available drug. Microfluidic nanofabrication outperforms traditional processes in terms of size distribution, batch consistency, and parameter controllability, and is naturally adapted to the rapid preparation requirements of small batches and multiple specifications. Recent reviews and methodological studies have shown that the microfluidic platform can output a nanoparticle library with size gradients and adjustable ligand density within a short time, and couple it with online characterization and algorithm optimization, thus approaching the turnaround-level individualized production capacity. This opens an engineering channel for future near-bedside preparation (point-of-care) and on-week medication [232,233].

During the treatment process, the most valuable realization of individualization is dynamic adaptation. MR-Linac makes the current imaging—current planning—current irradiation a reality [234]; when the nanoparticles themselves are MRI positive contrast agents, the amount and spatial distribution of the nanoparticles entering the tumor can be quantified and incorporated into the plan optimization, from dose carving to functional re-sketching, all based on the actual drug distribution rather than empirical assumptions. The updated research on adaptive radiotherapy (ART) and dose painting by numbers is advancing the coupling of imaging—dose—response to a multi-center replicable level [235,236]. Further, AI/Radiomics can input the current MRI/PET and previous molecular characteristics into the decision model to give executable recommendations such as whether to add nanoparticle drugs, whether to change the formulation, whether to combine with immunotherapy or chemotherapy, enabling the plan to evolve with the tumor [237,238].

The material dimension also allows for on-demand fine-tuning. For instance, in the mid-term assessment of hypoxic expansion, a nano-cerium system with a reversible Ce^3+^/Ce^4+^ cycle mechanism was introduced to enhance peroxide activation and ROS amplification [239,240]; while in normal tissue under physiological oxygen/normally acidic pH conditions, the nano-cerium tended to exhibit an antioxidant buffering effect, potentially reducing the toxicity to adjacent tissues. This scenario-dependent redox bidirectionality provides a mechanistic basis for phased replacement or stacking of different lanthanide formulations, and is in line with individualized safety boundary management [241,242].

The consistent anchor point is the verifiability of the integrated diagnosis and treatment platform in clinical practice. Taking AGuIX as an example, the early trials not only provided evidence of safety and renal clearance, but also established a rudimentary workflow of imaging quantification—plan adjustment—efficacy readout in studies of brain metastases and body tumors, shifting the nanomedicine from black box sensitization to visualized sensitization. With ongoing randomized studies yielding results and real-world data accumulating, combined with rapid iterative processes of radiogenomics/organoids/microfluidics and AI-driven methods, individualized lanthanide-based nanosensitizers are expected to move from exploratory protocols to standardized clinical pathways: first, stratify based on companion diagnostics, then manufacture in small batches to address patient heterogeneity, implement treatment with MR-Linac and AI for online adaptation, respond to ecological niche shifts by material switching, and ultimately implement the most appropriate nanodose, the most appropriate irradiation, and the most appropriate combination for individual patients in specific fractions [243,244,245].

In other words, individualization is not simply adding nanomedicine to radiotherapy, but rather a closed loop of stratification—verification—manufacturing—implementation—and re-stratification to maximize the net benefit for patients. Thanks to the clinical visualization of the lanthanide platform, the continuously mature adaptive radiotherapy and AI, as well as the representative transformation accelerators such as organoids and microfluidics, precise radiotherapy is shifting from experience-driven to data/evidence-driven. 

Beyond technical advancement, dose-painting and fraction-reduction enabled by lanthanide nanotheranostics align with global sustainability targets, specifically UN Sustainable Development Goal 3 (Good Health and Well-being), by curbing unnecessary radiation exposure and healthcare resource use, a concept recently reinforced by sustainability-focused analyses in nuclear medicine and radiotherapy optimization [246].

Moving along this path, lanthanide-based nanosensitizers will bring the ideal of “tailoring to the individual” from concept and case studies to replicable, regulatory, and affordable daily practice.

## 9. Conclusions

This review outlines the entire chain progress of Ln-NPs as tumor radiosensitizers from mechanism–evidence–transformation. In summary, Ln-NPs exert their radiosensitizing effect through an integrated multi-scale mechanism. The high-Z core enables physical dose enhancement via photoelectric and Auger effects, which synergizes with chemical ROS generation and biological disruption of DNA repair and immune activation. Recent quantitative and simulation-experiment evidence further suggests that the real biological geometry and loading conditions will significantly modulate the mapping relationship between dose enhancement and biological effect, emphasizing the necessity of standardization in characterization and reproducible experimental design to calibrate DEF and efficacy reading (such as MIRIBEL minimum information standards).

Integrating diagnosis and treatment is a significant advantage of the lanthanide platform. The ultra-small molecule nanoplatforms based on gadolinium, such as AGuIX, have the visualization characteristics of positive T1 contrast, which can quantitatively enter and remain in tumors after administration, providing measurable anchors for functional re-delineation and dose carving; early clinical and follow-up studies of NANO-RAD have proved its safety, feasibility, and quantifiable distribution, and have explored the inclusion of drug distribution in the plan optimization using methods such as T1 mapping in the multi-center workflow. Correspondingly, the rise of MRI-Linac and online adaptive radiotherapy (MRgART) has made the closed loop of imaging at the time—planning at the time—irradiation at the time possible, laying the technical and methodological foundation for imaging—dose—response online individualized management.

In terms of transformation evidence, in addition to lanthanide-based platforms, non-lanthanide high-Z references also provide external benchmarks: Hafnium oxide nanoparticles NBTXR3 achieved the primary pathological endpoint in a randomized study of soft tissue sarcoma and increased the R0 margin rate, suggesting that high-Z nanoradiotherapy sensitization has replicable clinical value and an assessable path, providing an important reference for the subsequent randomized design, endpoint selection, and regulatory communication of the lanthanide platform. At the same time, the heterogeneity of delivery and accumulation in the real world remains a common obstacle for nanomedicines, and multiple meta-analyses have shown that the median proportion of nanomedicines entering solid tumors after systemic administration is approximately 0.7% of the dose, highlighting the population differences caused by the EPR effect and the problem of cold areas within tumors, which require patient stratification and process/material optimization to address.

For the next step of clinical transformation, priority can be given to forming synergy around the four Cs: The first is manufacturability, relying on continuous flow and microfluidics for parallel in-line characterization, maintaining consistency in size, morphology, and surface chemistry from small-scale trials to large-scale production, while using data-driven formulation optimization to reduce costs and improve batch-to-batch stability; the second is safety, incorporating degradability, excretion, and traceability into the design, establishing a parameter set for pharmacokinetic and toxicology parameters such as dose—exposure—clearance, and through standardized follow-up to assess long-term accumulation and individual risk; the third is precision, using prospective stratification, companion diagnostics, and adaptive trial design to apply the appropriate nanomaterial formulation to the most likely beneficiaries and form a closed loop update in treatment with MRgART and quantitative imaging; the fourth is review, promoting the implementation of reporting standards such as MIRIBEL in characterization and biological assessment, and aligning with regulatory guidelines, using comparable and reproducible data packages to accelerate review and multi-center extrapolation. As the engineering, medical and regulatory elements are gradually aligned, lanthanide-based nanosensitizers are expected to transform “visualized sensitization and individualized dose management” from an exploratory concept into replicable clinical practice. This will enhance the local control rate while minimizing non-target tissue exposure and associated toxicity, ultimately converting the advantages of nanotechnology into tangible benefits for patients’ survival and quality of life.

## Figures and Tables

**Table 1:** Summary of major radiosensitisation mechanisms of lanthanide-based nanoparticles (Ln-NPs).

Mechanism Type	Key Process/Pathway	Representative Lanthanide Systems	Typical Evidence (Preclinical/Clinical)	References
Physical dose enhancement	High-Z-mediated photoelectric effect and Auger electron emission; nanoscale local dose amplification and clustered DNA DSBs	Gd_2_O_3_, NaGdF_4_-based NPs, Gd–Au hybrids	Monte Carlo simulations and in vitro/in vivo studies showing increased local dose and complex DNA damage patterns	[8,74]
ROS-mediated chemical sensitisation	Radiation-triggered or catalysed ROS (OH, O_2_^−^, H_2_O_2_) generation; oxidative damage to DNA, lipids, proteins	CeO_2_-based NPs, redox-active Ln oxides, Gd–Bi or Ln–Au hybrids	ROS assays, comet assays, increased oxidative stress and cell killing under irradiation	[36]
DNA repair inhibition	Interference with NHEJ/HR pathways; persistence of γ-H2AX foci; downregulation of BRCA1/RAD51 and related repair factors	Gd-based systems (AGuIX, Gd_2_O_3_ NPs)	Increased and sustained DNA damage foci; reduced clonogenic survival; altered DNA repair and apoptosis markers	[37,38,39]
Hypoxia-adaptive sensitisation	O_2_-independent ROS generation or H_2_O_2_ activation; maintained/enhanced effect in hypoxic & acidic TME	CeO_2_ nanozymes, Gd–Bi and related composites	Hypoxic tumor models showing sustained ROS production and radiosensitisation	[56,57,58,59]
Immunomodulatory synergy	Promotion of immunogenic cell death; activation of cGAS–STING and effector T cells; synergy with ICIs	Gd-based NPs + RT ± anti-PD-1/PD-L1	Preclinical models showing reinforced anti-tumour immunity and abscopal-like responses	[70,71]

**Table 2:** Representative lanthanide-based nanoradiosensitisers and their translational status.

Nanoplatform	Core Composition/Design	Key Functions	Stage/Model	Main Findings	References
AGuIX	Ultrasmall Gd-based polysiloxane/DOTA nanoplatform (<5 nm)	T_1_-weighted MRI contrast; pan-cancer radiosensitiser; renal clearance	Phase I (NANO-RAD), Ib/II (NANO-GBM), multiple Phase II/MR-Linac trials ongoing	Safe IV administration; MRI-visible tumour uptake; supports “visualised sensitisation” and dose-painting, image–dose coupling	[61,62,63,64,92,93]
AGuIX-Bi	Bi-doped Gd-based theranostic nanoplatform	High-Z (Gd/Bi) synergy; enhanced secondary electron yield; MRI-visible	Preclinical xenograft models	Stronger radiosensitisation vs. AGuIX, delayed tumour growth, ~33% CR in reported model; maintains imaging capability	[63,91]
Gd_2_O_3_@BSA–Au	Core–shell/hybrid Gd_2_O_3_–Au nanostructures stabilised by biomacromolecules	Dual/multimodal imaging (MRI/CT); physical dose enhancement + ROS amplification	Preclinical	Increased ROS, reduced clonogenic survival, improved tumour control with acceptable toxicity; showcases “physical + chemical” synergy	[101,102]
CeO_2_-based NPs	Redox-active Ce^3+^/Ce^4+^ nanozymes	Context-dependent ROS modulation; potential hypoxia-tolerant radiosensitisation	Preclinical in vitro/in vivo	Maintain or adapt ROS-related effects in tumour-like hypoxia; potential tumour-selective sensitisation with normal-tissue protection	[85,86,87]
Gd–Au and other Ln–high-Z hybrids	Alloyed or core–shell composites combining lanthanides with Au, Bi, etc.	High-Z synergistic enhancement; MRI + CT/optical multimodal imaging; theranostic potential	Simulation + preclinical	Higher DEF via multi-element synergy; flexible imaging; promising for integrated diagnosis–treatment–immunomodulation strategies	[98,99,100,101,102]

**Table 3:** Representative radiosensitization indicators for lanthanide-based nanomaterial systems under different irradiation conditions.

Nanomaterial System	Reported Radiosensitization Indicator	Energy Regime/Condition	Notes	References
AGuIX (Gd-based)	Sensitization Enhancement Ratio (SER)	Low keV (e.g., <150 keV)	Significant SER reported, e.g., ≈1.1–2.5 in vitro studies	[113]
AGuIX (Gd-based)	Radiosensitization observed	MV clinical beams	Enhancement often modest; literature describes less pronounced effects at high energy	[3]
Gd_2_O_3_/Gd-oxide NPs	Dose enhancement/ROS increase	Low-energy irradiation	Reported enhancement of ROS production; maximum ~1.15–1.94 reported	[114]
Bi-containing high-Z NPs	Average DEF ~1.4–1.5 (meta-analysis)	Preclinical studies	Meta-analysis shows mean DEF ~1.41–1.47 across studies	[115]
CeO_2_ and other lanthanide oxides	Radiosensitization observed	Various	Specific quantitative ranges not widely reported across energies	[116]

## Data Availability

Data availability is not applicable to this article as no new data were created or analyzed in this study.
